# Pulmonary sclerosing pneumocytoma masquerading as lung metastasis: A case report

**DOI:** 10.1097/MD.0000000000050338

**Published:** 2026-08-28

**Authors:** Mi Young Kim, Hee Yong Kwak, Hojung Lee, Man Sil Park

**Affiliations:** a Department of Radiology, Konkuk University Medical Center, Konkuk University School of Medicine, Seoul, Republic of Korea; b Department of Surgery, Nowon Eulji University Hospital, Eulji University School of Medicine, Seoul, Republic of Korea; c Department of Pathology, Nowon Eulji University Hospital, Eulji University School of Medicine, Seoul, Republic of Korea; d Department of Thoracic and Cardiovascular Surgery, Nowon Eulji University Hospital, Eulji University School of Medicine, Seoul, Republic of Korea.

**Keywords:** computed tomography scan, pulmonary sclerosing pneumocytoma, thyroid transcription factor-1

## Abstract

**Rationale:**

Pulmonary sclerosing pneumocytoma (PSP) is a rare benign neoplasm primarily affecting middle-aged Asian women. Although typically presenting as a well-circumscribed solitary nodule with an indolent clinical course, PSP can occasionally exhibit aggressive radiological features. In patients with a history of malignancy, these atypical findings can lead to a significant diagnostic dilemma, frequently mimicking metastatic disease.

**Patient concerns:**

A 42-year-old female with a history of breast cancer was referred for evaluation of a lung nodule identified during surveillance. Contrast-enhanced chest computed tomography (CT) revealed a 1.6-cm juxtapleural mass in the right middle lobe. Notably, the lesion showed apparent vessel abutment and suspected vascular invasion, leading to a presumptive radiological diagnosis of angioinvasive metastasis. F-fluorodeoxyglucose positron emission tomography/CT demonstrated low F-fluorodeoxyglucose uptake with a maximum standardized uptake value of 1.6.

**Diagnoses:**

Despite the preoperative suspicion of malignancy based on aggressive CT features, the diagnosis of PSP was confirmed through histopathological examination following surgical intervention.

**Interventions:**

The patient underwent an open wedge resection. Intraoperative findings revealed a well-demarcated mass without inflammatory adhesions or pericardial involvement.

**Outcomes:**

The postoperative course was uneventful, and no recurrence was observed during the follow-up period.

**Lessons:**

This case highlights that PSP can radiologically masquerade as angioinvasive metastasis, especially in oncological patients. Clinicians and radiologists should consider PSP as a differential diagnosis for solitary pulmonary nodules with suspected vascular invasion, even when positron emission tomography/CT shows low metabolic activity. Histopathological confirmation remains the gold standard for avoiding overdiagnosis and unnecessary radical treatment.

## 1. Introduction

The identification of a solitary pulmonary nodule during the staging of breast cancer represents a critical clinical dilemma. Such a finding is often prioritized as distant metastasis, which upstages the malignancy to Stage IV. This diagnosis necessitates a shift from curative-intent surgery to palliative systemic therapy, fundamentally altering the patient’s prognosis. However, the lung can also harbor rare benign neoplasms that radiologically mimic malignancy.

Among these, pulmonary sclerosing pneumocytoma (PSP) stands out as a “great mimicker.” Originally thought to be vascular in origin – hence the former term “sclerosing hemangioma” – this rare entity is now understood to be epithelial in nature, as reflected in its reclassification in the 2015 World Health Organization system.^[1]^ Radiologically, PSP typically presents as a solitary, well-circumscribed nodule in juxtapleural or juxtafissural locations, often characterized by distinct computed tomography (CT) signs such as a marginal pseudocapsule (50%), overlying vessels (26.3%), halo signs (17.1%), and occasionally air gaps (2.6%).^[2]^ Nevertheless, these radiological features are largely nonspecific, posing significant challenges for definitive preoperative diagnosis.

In clinical contexts involving primary malignancies, the nonspecific radiological nature of PSP can lead to significant diagnostic pitfalls. Such ambiguity may result in suboptimal therapeutic stratification, where a potentially resectable benign lesion is mismanaged as advanced metastatic disease. We report a case of a 42-year-old female with breast cancer in whom PSP was initially interpreted as lung metastasis. This case highlights the clinical importance of pursuing histopathologic confirmation to ensure accurate pathological staging and the implementation of appropriate therapeutic interventions, even when imaging findings are suggestive of malignancy.

## 2. Case presentation

**Figure 1.**
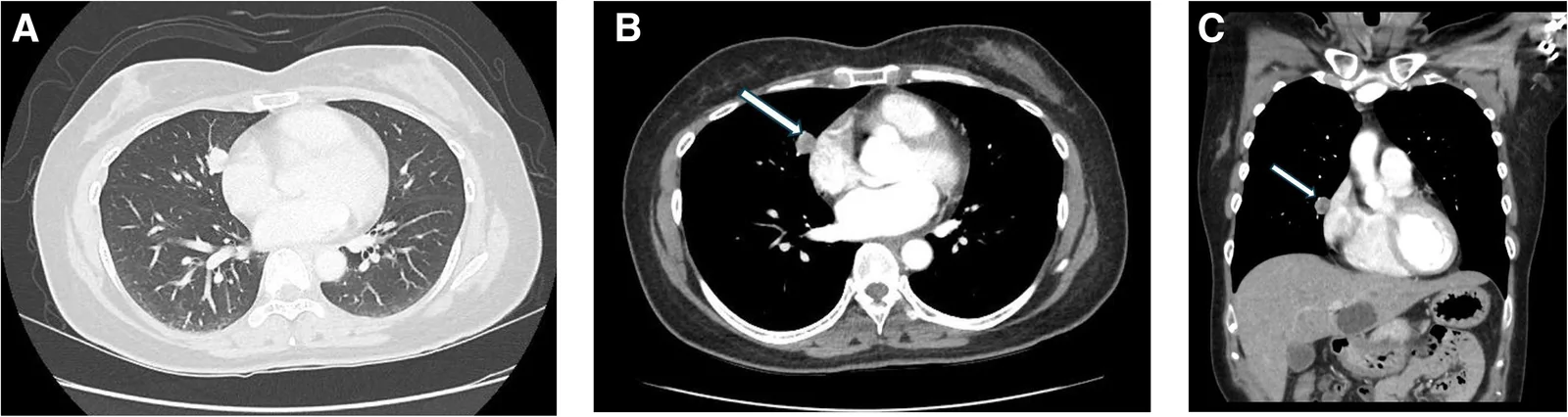
(A–C) Chest CT demonstrated a 1.6-cm, well-defined solid nodule (arrow) with inhomogeneous enhancement in the medial segment of the right middle lobe, immediately adjacent to the mediastinal pleura. Note the loss of the intervening fat plane between the lesion and adjacent pulmonary vasculature. CT = computed tomography.

**Figure 2.**
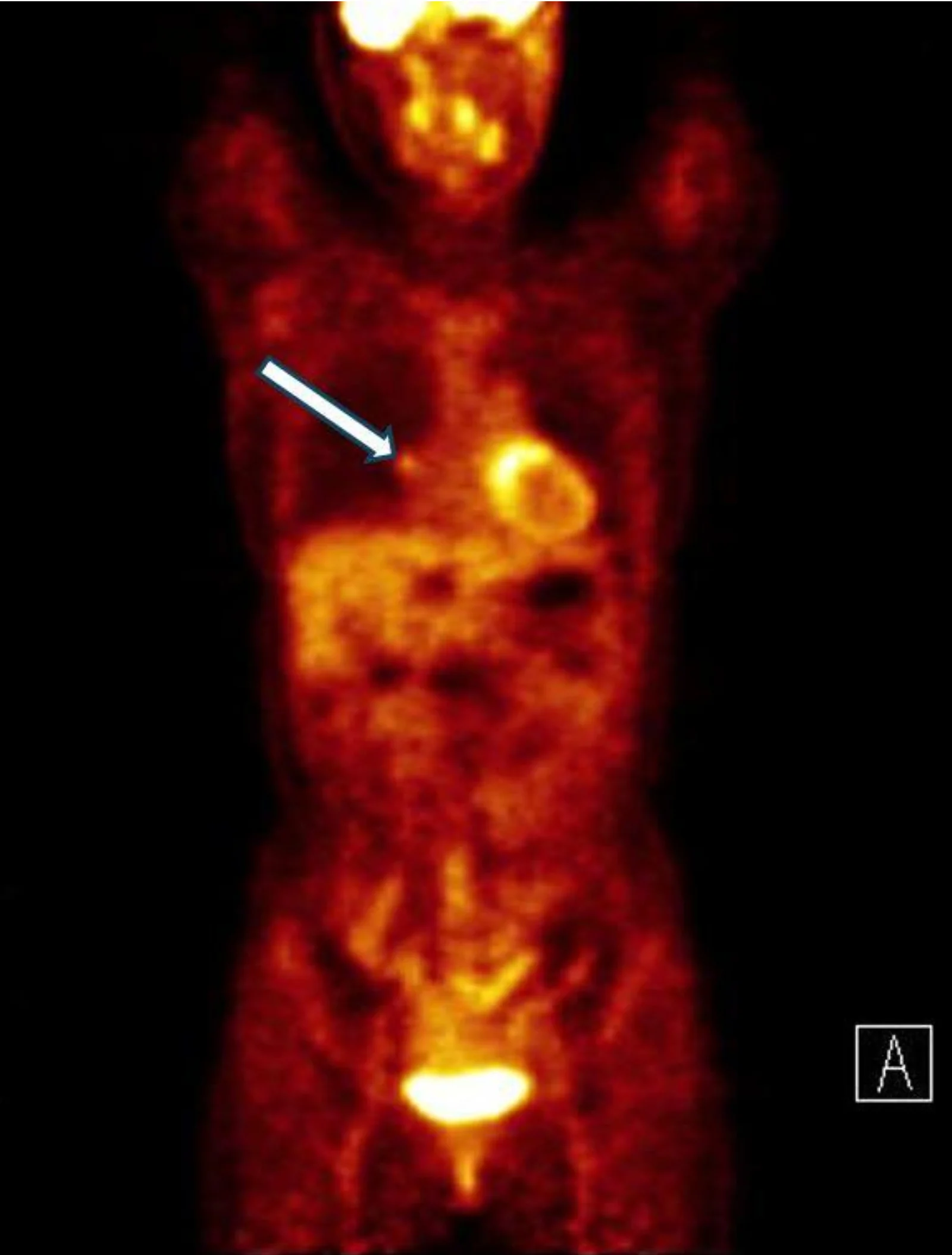
PET/CT showed a nodule (arrow) with mild FDG uptake with a maximum standardized uptake value (SUVmax) of 1.6 in the right middle lobe. CT = computed tomography, FDG = F-fluorodeoxyglucose, PET = positron emission tomography.

**Figure 3.**
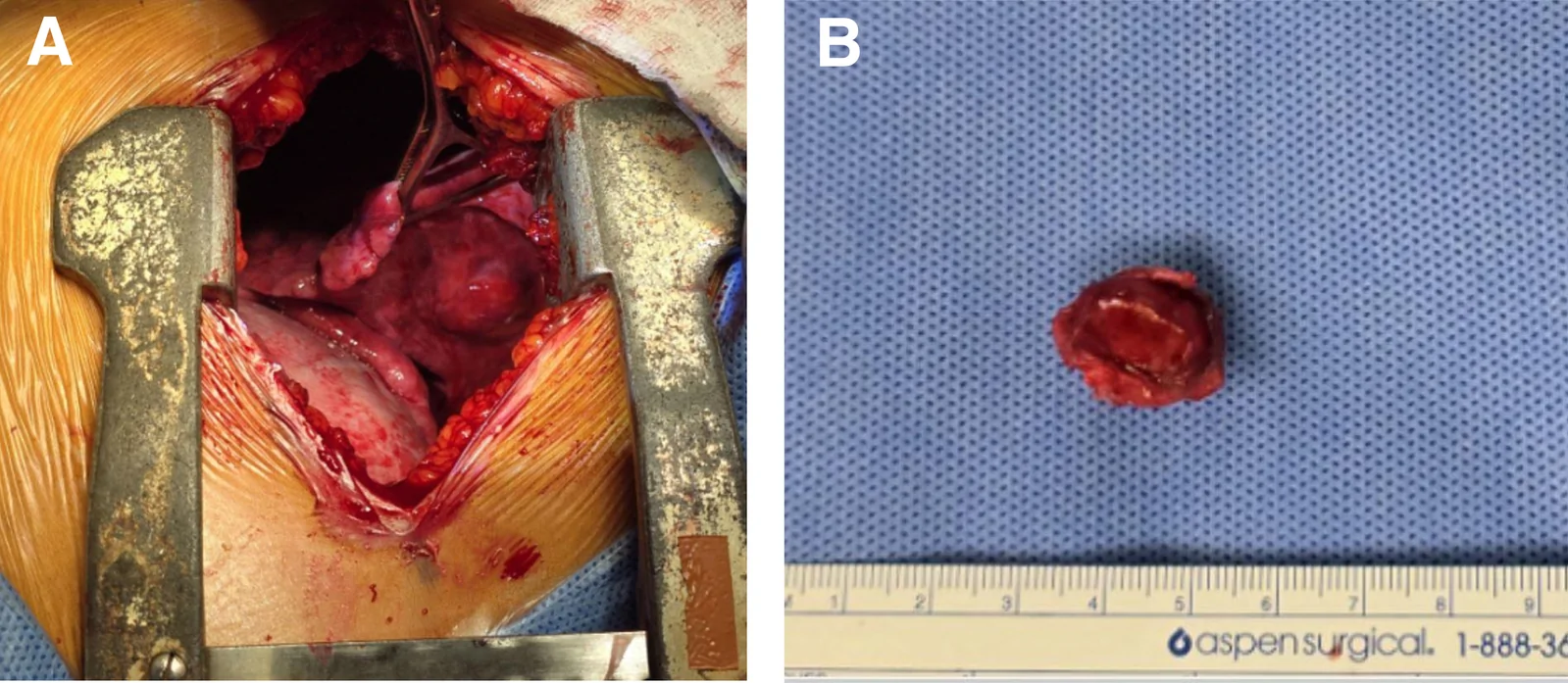
Intraoperative finding. A well-demarcated mass (A) is observed in the medial segment of the right middle lobe, closely abutting the pericardium (B).

**Figure 4.**
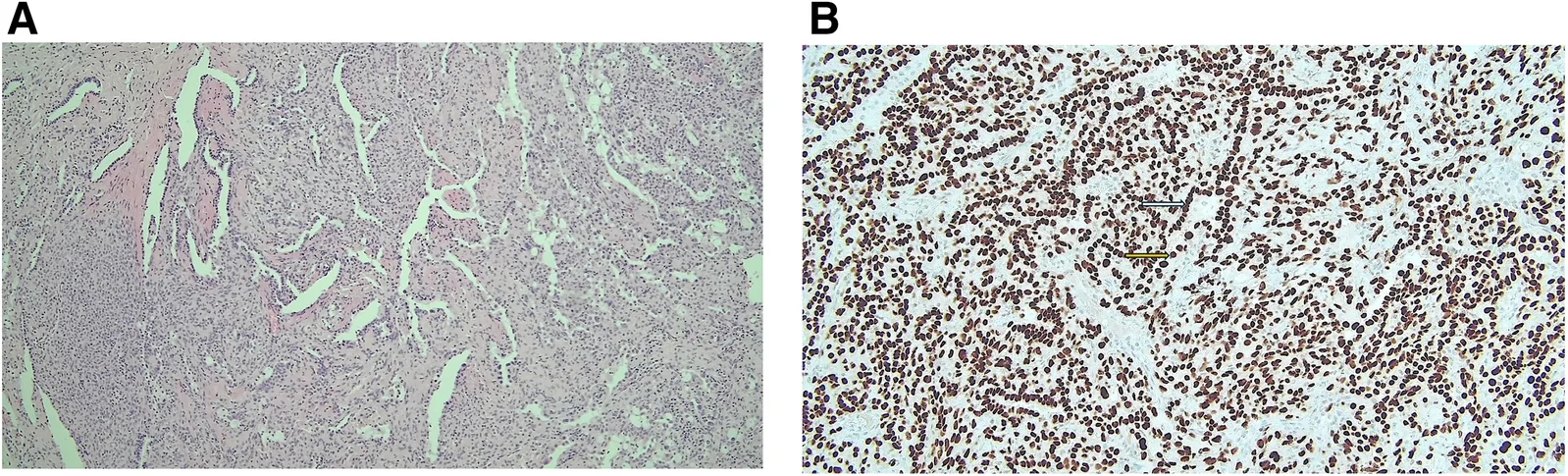
Pathologic findings of pulmonary sclerosing pneumocytoma (A) The tumor is composed of 2 cell types: cuboidal surface cells and round stromal cells, showing papillary and sclerotic growth pattern (H&E, X100). (B) The surface cells (white arrow) and round stromal cells (yellow arrow) are positive for TTF-1 (thyroid transcription factor-1) immunohistochemical staining (X200).

This study was approved by the Institutional Review Board of Nowon Eulji University Hospital, and the requirement for informed consent was waived due to the retrospective and anonymous nature of the case report (IRB No. 2026-03-018). A 42-year-old female presented to our institution in August 2024 for the evaluation of a palpable mass in her right breast. Breast ultrasonography identified a 2 cm, irregular-shaped, hypoechoic mass with a microlobulated margin at the 12 o’clock position of the right breast and a subsequent ultrasound-guided core needle biopsy confirmed invasive ductal carcinoma. For initial staging, a contrast-enhanced chest CT was performed, revealing a 1.6 cm, well-defined solid nodule with inhomogeneous enhancement in the medial segment of the right middle lobe, immediately adjacent to the mediastinal pleura (Fig. 1A–C). Notably, the lesion exhibited a loss of the intervening fat plane with adjacent pulmonary vascular structures. Due to this suspected vascular invasion, the nodule was radiologically interpreted as angioinvasive metastasis, considering the patient’s concurrent diagnosis of invasive ductal carcinoma of the breast. A preoperative percutaneous needle biopsy was considered for differentiation but was deemed technically unfeasible because the lesion was located in the immediate vicinity of the pericardium. Further evaluation with F-fluorodeoxyglucose (FDG) positron emission tomography (PET)/CT was delayed due to an institutional equipment malfunction. During this period, the primary breast cancer exhibited rapid clinical enlargement. After a thorough shared decision-making process, the patient underwent urgent breast-conserving surgery to prioritize the primary malignancy. In the postoperative state, a subsequent PET/CT was performed, which showed a nodule with mild FDG activity with a maximum standardized uptake value (SUVmax) of 1.6 (Fig. 2). Although the metabolic activity was relatively low, the persistent suspicion of vascular invasion precluded the exclusion of distant metastasis. Consequently, rather than a video-assisted thoracic surgery (VATS), the patient underwent an open mini-thoracotomy with wedge resection for a definitive diagnosis. Because the tumor was located in the medial segment of the right middle lobe, performing a standard VATS wedge resection carried a risk of unnecessarily removing too much normal lung parenchyma. Furthermore, while a VATS approach would require inserting 3 trocars and potentially expanding an incision to extract the tumor, a small open thoracotomy was chosen to enucleate the mass precisely and preserve as much normal lung parenchyma as possible. Intraoperative findings showed a well-demarcated mass that was located in the medial segment of the right middle lobe (Fig. 3A and B). Despite its close proximity to the pericardium, the lesion showed no evidence of inflammatory adhesions or direct invasion. Microscopically, the tumor was composed of 2 cell types: cuboidal surface cells and round stromal cells, showing a papillary and sclerotic growth pattern (Fig. 4A). No hemorrhage, necrosis, or mitotic figures were noted. Immunohistochemical stains were positive for cytokeratin (CK) and thyroid transcription factor-1 (TTF-1), faintly positive for CD31, focally positive for estrogen receptor (ER), and negative for D2-40 (Fig. 4B). Based on these histological and immunohistochemical findings, the tumor was diagnosed as PSP. Regarding the focal ER positivity in our patient, the immunoreactivity was subtle and weak in intensity. Notably, this presentation of weak ER expression is supported by the literature of Devouassoux-Shisheboran et al, which reported that approximately 7% of the PSP cases subjected to immunohistochemical staining exhibited a similarly weak ER positivity.^[3]^ Furthermore, given that the patient’s underlying primary breast cancer was a strongly ER-positive case with an Allred score of 8, this immunophenotypic mismatch – specifically the discordance in receptor expression intensity – significantly decreases the likelihood of metastasis from the known breast cancer. In conjunction with the distinct positivity for TTF-1, a specific marker of pneumocytic origin, the probability of metastatic breast carcinoma is further diminished, thereby confirming the diagnosis of PSP. The surgery ended with an open wedge resection alone. While the patient remained on her ongoing treatment regimen for breast cancer, no additional treatment was required for the PSP following its complete excision.

## 3. Discussion

Evolving from Liebow and Hubbell original classification as a vascular lesion,^[4]^ PSP is now established as a benign neoplasm arising from primitive respiratory epithelium expressing TTF-1.^[3]^ Accordingly, the 2015 World Health Organization classification reclassified PSP into the “Adenomas” group, moving it from the “Miscellaneous tumors” category of previous editions.^[1]^ Histologically, PSP is characterized by a distinctive dual cell population consisting of surface cells similar to type II pneumocytes and round cells, with marginally different histogenetic profiles.^[1]^ Immunohistochemically, the surface cells are positive for both TTF-1 and cytokeratin, whereas the round cells are characteristically positive only for TTF-1.^[3,5]^ These cells are arranged in a mixture of 4 architectural patterns: papillary, sclerotic, solid, and hemorrhagic.^[6–8]^ Literature indicates that a combination of all 4 patterns is most common (50.0–76.9%), followed by 3 patterns (19.2–43.8%), while a two-pattern combination represents a relatively small percentage, and single-pattern tumors are exceedingly rare.^[3,7]^ Consistent with these established criteria, our patient’s tumor microscopically demonstrated a less frequent two-pattern combination consisting of predominant papillary and sclerotic growth, notably without any hemorrhage, necrosis, or mitotic figures. Because of this architectural diversity and histological overlapping, the diagnostic process for PSP can be critically challenging in frozen sections, small needle biopsies, or cytology, where it can be confused with primary adenocarcinoma or carcinoid tumors.^[1,6]^ PSP typically has a benign clinical course, with metastasis being exceedingly rare.^[3]^ While the primary treatment for PSP is surgical resection, the recurrence rate remains negligible, ranging from 0 to 3.8% over retrospective follow-up periods spanning 1 to 228 months.^[2,3,9,10]^

To the best of our knowledge, there are limited reports documenting PSP identified during the initial staging workup of a primary malignancy where the clinico-radiological suspicion of metastasis was high. While Luca et al described a rare instance of PSP mimicking lung metastasis, our case is distinguished by even more aggressive radiological features.^[11]^ The presence of suspected vascular invasion in our patient suggested a likelihood of angioinvasive malignancy. This underscores that PSP can present with highly deceptive, invasive-looking indicators that pose a significant diagnostic challenge. This case highlights a significant diagnostic pitfall: even in oncological patients where distant dissemination is clinically presumed, benign mimics like PSP must remain in the differential diagnosis to ensure accurate pathological staging and optimal treatment planning. In our case, the clinical and radiological findings – specifically the suspicious vascular involvement and the patient’s concurrent aggressive breast cancer – strongly favored a diagnosis of solitary lung metastasis.

PSP is characterized by a female predilection, with the female-to-male ratio typically ranging from 5:1 to 7.7:1.^[2,12]^ Furthermore, some studies indicate that this female predominance is even more pronounced in Asian populations.^[6,13,14]^ Clinically PSP is primarily asymptomatic, with reports indicating that 92.1 to 96.6% of cases are incidentally detected.^[2,3,12,15]^ These lesions are typically discovered during routine health screenings or through follow-up imaging for unrelated respiratory conditions. In our case, while the patient’s demographic profile – a 42-year-old female – aligns with the typical peak incidence of PSP, the clinical context of a concurrent primary malignancy significantly confounded the initial diagnosis. Given the high-risk setting of newly diagnosed breast cancer, the possibility of a solitary pulmonary metastasis remained a primary concern that could not be excluded without histopathological confirmation.

On conventional CT scans, PSP typically presents as a well-defined juxtapleural or perihilar mass, exhibiting inhomogeneous enhancement (67–157 HU) that may reflects its diverse histopathological architecture, including solid, papillary, angiomatous, and sclerotic components.^[2]^ The CT signs included marginal pseudocapsule (50%), overlying vessel (26.3%), air gap (2.6%), and halo sign (17.1%). While PSP predominantly presents as a solitary lesion (ranging from 96.0 to 100% in various series), its lobar distribution varies across studies, although it is most frequently identified in the left lower lobe.^[10,12]^ In our case, the lesion manifested as a 1.6 cm well-defined solid nodule with inhomogeneous enhancement in the medial segment of the right middle lobe, intimately abutting the mediastinal pleura. Although the juxtapleural location was consistent with typical PSP, the absence of other characteristic CT findings – combined with suspected vascular invasion – led to a clinical prioritization of angioinvasive metastasis over a benign entity. Specifically, in the high-risk setting of newly diagnosed invasive breast cancer like our case, this suspicious vascular involvement, combined with the otherwise well-defined morphology, led us to clinically prioritize a solitary angioinvasive metastasis over a primary pulmonary malignancy. From a differential diagnostic standpoint, a peripheral solitary pulmonary nodule with well-circumscribed morphology requires the consideration of pulmonary carcinoid tumors and other benign nodules.^[16–18]^ When located peripherally, carcinoid tumors characteristically present as well-defined lobulated nodules or masses with intense contrast enhancement; this features distinguishes them from the inhomogeneous enhancement observed in our case, which reflects the mixed histological components of PSP. Additionally, other benign nodules, such as hamartomas or granulomas, can be considered as they manifest as smooth, well-demarcated solid masses. Nevertheless, these benign entities often display specific radiological hallmarks, such as internal fat attenuation or characteristic calcifications, which were absent in our case. Ultimately, the lack of these benign indicators, combined with the suspicious vascular involvement, created a diagnostic pitfall that led to the lesion being mistaken for distant metastasis, potentially resulting in clinical overstaging. Furthermore, the lesion’s close proximity to the pericardium rendered a preoperative biopsy infeasible, further contributing to the diagnostic uncertainty. While VATS is widely established as the standard minimally invasive approach for peripheral lung nodules, an open mini-thoracotomy with wedge resection was intentionally selected for our patient. Because the tumor was located deep within the medial segment of the right middle lobe, a standard VATS wedge resection carried a significant risk of unnecessarily sacrificing an excessive volume of normal lung parenchyma. Furthermore, whereas a conventional VATS approach would necessitate multiple trocar insertions and a potential secondary incision extension for specimen extraction, a meticulous open mini-thoracotomy allowed the surgical team to precisely enucleate the mass. This tactical decision minimized parenchymal damage and maximized tissue preservation while ensuring immediate control against the suspected vascular invasion. Crucially, histopathological and immunohistochemical evaluations provided the definitive resolution to this diagnostic dilemma, ruling out metastatic breast carcinoma. Although our patient’s nodule exhibited focal ER positivity, the immunoreactivity was remarkably subtle and weak in intensity. According to Devouassoux-Shisheboran et al,^[3]^ such weak, focal ER expression occurs in approximately 7% of PSP cases. This immunophenotypic profile presents a s contrast to the patient’s primary breast malignancy, which was a strongly ER-positive invasive ductal carcinoma of the breast with an Allred score of 8. This significant discordance in receptor expression intensity, combined with the distinct positivity for TTF-1 – a specific marker of pneumocytic origin – conclusively excluded the possibility of a metastatic lesion, thereby establishing the final diagnosis of PSP.

Literature indicates that PSP typically exhibits low-to-intermediate FDG avidity; apart from a few exceptional cases, the reported mean SUVmax is 2.96 ± 1.88.^[19–21]^ Although our case presented with a relatively low SUVmax of 1.6, this hypometabolic uptake could not definitively exclude metastatic disease given the presence of a concurrent primary malignancy. Therefore, when radiological features of PSP are atypical or suspicious for vascular invasion, surgical confirmation remains essential to prevent clinical understaging of a potentially metastatic cancer.

## 4. Conclusions

This case of PSP highlights the difficulty in diagnosing this rare tumor. Although a solitary pulmonary nodule identified during the staging workup of a primary malignancy is frequently prioritized as metastasis, the possibility of benign mimics such as PSP should be considered in the differential diagnosis. Recognizing PSP as a potential diagnostic pitfall is essential to avoid clinical overstaging and to ensure that patients are not inadvertently excluded from curative surgical opportunities for their primary cancer. Therefore, maintaining a high index of suspicion for such benign entities during the diagnostic workup is fundamental to determining the optimal treatment plan.

## Author Contributions

**Conceptualization:** Mi Young Kim, Hee Yong Kwak.

**Data curation:** Mi Young Kim, Hee Yong Kwak, Hojung Lee, Man Sil Park.

**Formal analysis:** Mi Young Kim, Hee Yong Kwak, Man Sil Park.

**Investigation:** Mi Young Kim, Hee Yong Kwak, Hojung Lee, Man Sil Park.

**Methodology:** Mi Young Kim, Hee Yong Kwak, Hojung Lee, Man Sil Park.

**Project administration:** Mi Young Kim, Hee Yong Kwak.

**Resources:** Mi Young Kim, Hee Yong Kwak, Hojung Lee, Man Sil Park.

**Software:** Hee Yong Kwak.

**Supervision:** Mi Young Kim, Hee Yong Kwak.

**Validation:** Mi Young Kim, Hee Yong Kwak, Man Sil Park.

**Visualization:** Mi Young Kim, Hee Yong Kwak, Hojung Lee, Man Sil Park.

**Writing – original draft:** Mi Young Kim, Hee Yong Kwak, Hojung Lee, Man Sil Park.

**Writing – review & editing:** Mi Young Kim, Hee Yong Kwak, Hojung Lee, Man Sil Park.
